# Associations Between Smartphone-Based Finger Tapping and Cognitive Performance in Older Adults: Observational Study

**DOI:** 10.2196/82463

**Published:** 2026-02-18

**Authors:** Huitong Ding, Taylor A Orwig, Jian Rong, Heaven Y Tatere, Chunyu Liu, Eric Schramm, Chathurangi H Pathiravasan, Joanne M Murabito, Honghuang Lin

**Affiliations:** 1 Department of Anatomy and Neurobiology, Boston University Chobanian & Avedisian School of Medicine, Boston, MA, United States; 2 The Framingham Heart Study, Boston University Chobanian & Avedisian School of Medicine, Boston, MA, United States; 3 Department of Medicine, University of Massachusetts Chan Medical School, 55 Lake Avenue North, Worcester, MA, 01655, United States, 1 774 455 4881; 4 Department of Neurology, Boston University Chobanian & Avedisian School of Medicine, Boston, MA, United States; 5 Department of Biostatistics, Boston University School of Public Health, Boston, MA, United States; 6 Care Evolution, Ann Arbor, MI, United States; 7 Department of Biostatistics, Johns Hopkins Bloomberg School of Public Health, Baltimore, MD, United States; 8 Department of Medicine, Section of General Internal Medicine, Boston University Chobanian & Avedisian School of Medicine, Boston, MA, United States

**Keywords:** finger tapping test, smartphone, digital features, cognitive performance, cohort

## Abstract

**Background:**

Finger tapping tasks assess fine motor control and have been proposed as potential markers of cognitive function. With smartphones widely available, these tasks can be easily administered at home or in other nonclinical settings. However, the relationship between smartphone-based finger tapping measures and cognitive performance is still not well understood.

**Objective:**

This study aimed to examine the association between smartphone digital finger tapping features and cognitive performance in an aging population.

**Methods:**

Participants were enrolled in the study as part of the electronic Framingham Heart Study. They were instructed to perform a 2-finger tapping task every 2 months over a 1-year period. In each session, they tapped with 2 fingers of the left hand for 10 seconds and then with 2 fingers of the right hand for 10 seconds, for a total of 20 seconds. Global cognition performance and 4 cognitive domains, including memory, executive function, language, and visuospatial function, were assessed using a standardized neuropsychological battery. Fifteen tapping features were extracted to capture aspects of motor performance, including the mean, SD, skewness, and slope of the intertap interval (ITI). Associations between tapping-derived features and cognitive performance were assessed using linear regression models, adjusting for age, sex, handedness, education, cohort, and the time interval between cognitive and tapping assessments.

**Results:**

A total of 302 participants (mean age 74.7, SD 6.3 years; n=169, 56% female participants, and n=40, 13.2% non-White participants) completed the digital finger tapping tasks. Eleven tapping features such as basic temporal properties (eg, number of taps and mean ITI), temporal variability (eg, SD and coefficient of variation of ITI, ITI range, and Microfluctuation Index), and fatigue/temporal drift (ITI slope) were significantly associated with global cognitive function (all *P*<.001). Each 1 SD increase in the number of taps was associated with a 0.14-unit higher global cognitive function score (*β*=.14, 95% CI 0.07-0.21). Furthermore, finger tapping features were significantly associated with multiple cognitive domains, with the greatest number of associations observed for executive function (11 significant features), including a strong association with mean ITI (*β*=−0.27, 95% CI −0.33 to −0.20). Stratified analyses by hand showed consistent effect directions across both hands. The aggregated composite scores derived from finger tapping features demonstrated significant associations with their respective cognitive test scores, with partial correlation coefficients ranging from 0.21 for memory function to 0.41 for global cognitive function.

**Conclusions:**

Digital finger tapping features, particularly those reflecting ITI variability, are significantly associated with cognitive performance. These findings suggest that finger tapping tasks may serve as noninvasive, scalable tools for assessing cognitive performance. Further research is warranted to validate their use for monitoring cognitive health in aging populations.

## Introduction

Motor performance has emerged as a promising indicator of cognitive aging. Growing evidence suggests its potential utility as an early marker of cognitive decline [1]. While gait-related metrics have been extensively explored and consistently associated with impairments in memory, executive function, and visuospatial function [2,3], there is increasing recognition that motor biomarkers should also include upper-limb function. Recent studies have suggested that abnormalities in upper-limb motor function, such as slower movement speed and irregular rhythm, may reflect early neurodegenerative changes and have been associated with mild cognitive impairment (MCI) and dementia [4-6].

Among upper-limb assessments, the finger tapping test (FTT) is a simple and widely used measure of psychomotor speed, motor coordination, and central nervous system integrity [4,7]. Early versions of the FTT used a telegraph key device [8]. Later adaptations moved to devices such as the computer mouse [9] and keyboard [10]. With the widespread availability of smartphones, the FTT can now be administered on touchscreen interfaces [11,12]. This approach is low-cost and scalable. And it allows fine motor function to be assessed outside traditional clinical settings. Smartphones are also well-suited for population-level digital phenotyping because they can support at-home assessments that are more convenient and accessible for diverse and underserved groups. In addition, digital platforms capture high-resolution temporal data. This makes it possible to derive a wide range of tapping features that may help identify subtle motor changes related to early neurodegenerative processes.

Neuropsychological testing remains the gold standard for assessing domain-specific cognitive performance including memory and executive function [13]. However, high-resolution digital motor measures may provide complementary information about brain-behavior relationships. Examining how fine motor measures derived from widely accessible technologies relate to cognitive performance could improve the way we characterize cognitive aging and support the development of scalable digital biomarkers. Linking accessible, real-world digital tools with rigorous neuropsychological assessment is an important step toward more precise evaluation of cognitive function in older adults and may ultimately contribute to earlier identification of subtle changes.

In this study, we examined the relationship between smartphone-based finger tapping performance and multiple cognitive domains in participants from the Framingham Heart Study (FHS), a well-characterized multigenerational cohort. We hypothesized that poorer tapping performance, as captured by digital tapping-derived metrics, would be associated with lower cognitive performance. By linking these digital motor measures with standard neuropsychological tests, our findings may help support the use of smartphone-based tapping tasks as scalable tools for characterizing cognitive function in aging.

## Methods

### Study Population

**Figure 1.**
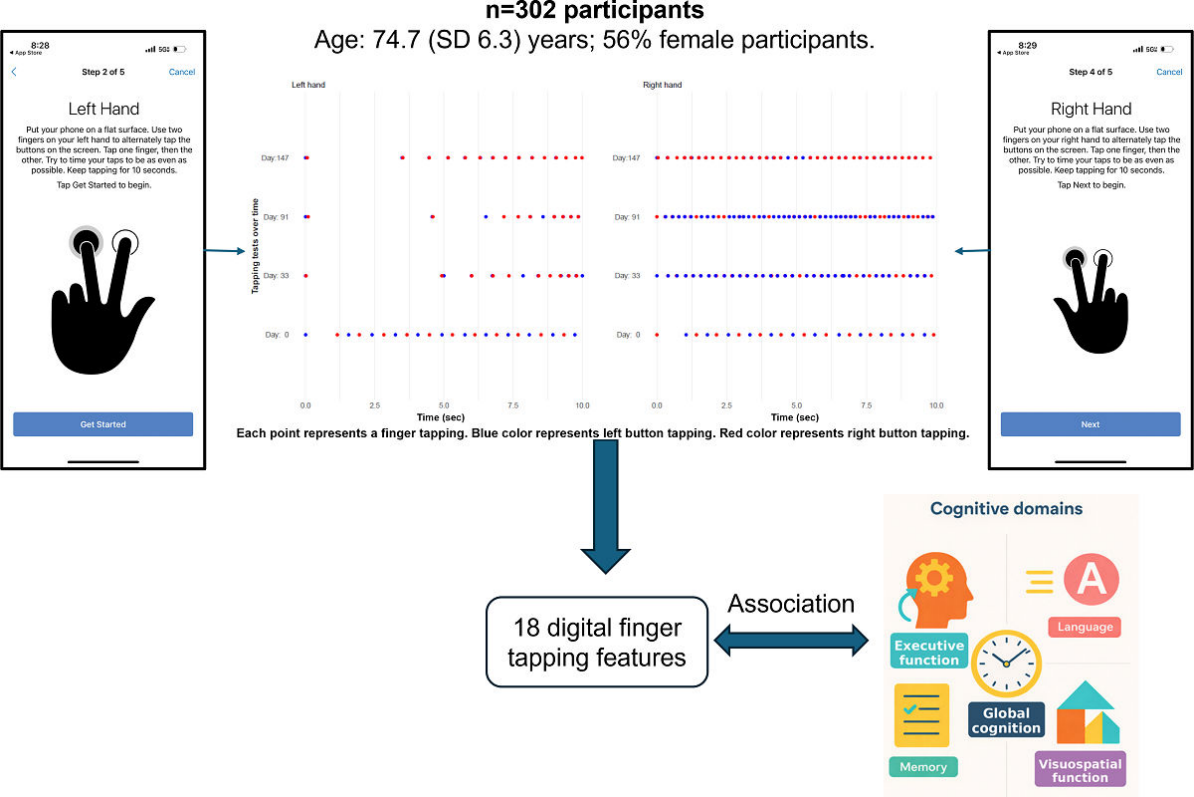
Graphical abstract.

This study included Offspring and Omni 1 cohort participants from the FHS, a longitudinal and multigenerational cohort study initiated in 1948 [14-16]. Offspring participants were enrolled from 1971 to 1975 (n=5124), and the multiethnic Omni 1 participants were enrolled from 1994 to 1998 (n=506) to reflect the increasing diversity of the city of Framingham, MA. Both cohorts were examined in the FHS research center every 4 to 8 years using the same protocols. Eligible participants were invited to enroll in the electronic FHS (eFHS) at the time of the last research exam (Offspring examination 10 and Omni 1 examination 5, 2019‐2022). English-speaking participants who owned a smartphone (iPhone with iOS 10 or higher or Android version 7 or higher) were invited to download the MyDataHelps app, which included a number of surveys and tasks. A research assistant assisted participants with app download in the research center and provided written instructions if participants preferred to download the app at home. Further details of the eFHS protocol have been previously described [17]. A total of 620 participants enrolled in the eFHS study. Of these, 9 participants who enrolled using a computer and 133 participants who enrolled using an Android device were excluded, as the 2-finger tapping test was only available on the iPhone platform. Among the remaining participants, 445 completed at least one 2-finger tapping test. For the present analysis, we further restricted the sample to individuals who had completed at least one 2-finger tapping assessment and at least one neuropsychological assessment within a 5-year window. This additional criterion yielded a final analytic sample of 302 participants. Participants provided electronic consent within the eFHS mobile app and gave written consent for the parent FHS at each in-person exam visit and neuropsychological testing session. Figure 1 presents the graphical abstract of this study.

Cognitive and clinical characteristics were assessed using standardized procedures. Cognitive status was adjudicated by a review committee that included at least 1 neurologist and 1 neuropsychologist [18]. Dementia was diagnosed according to the criteria outlined in the Diagnostic and Statistical Manual of Mental Disorders, Fourth Edition [19]. MCI was defined as cognitive decline in one or more domains without functional impairment and not meeting criteria for dementia [20]. Details of the FHS diagnostic procedures have been described in a prior study [21]. Parkinson disease (PD) status was determined using 3 sources: (1) self-report to the question “have you been told by a doctor you have PD?” (2) examiner-administered health history interviews; and (3) responses to the question “for what medical conditions do you use cannabis?” where PD was listed as an option. Self-rated health was measured using a question from the self-administered Short Form-12 Health Survey. In this survey, participants rated their overall health on a 4-point scale ranging from “poor” to “excellent” [22].

### Ethical Considerations

This research received ethical approval from the institutional review board of Boston University Medical Center (H-32132). Participants provided electronic consent within the eFHS mobile app and gave written consent for the parent FHS at each in-person exam visit and neuropsychological testing session. The original informed consent and institutional review board approval allowed secondary analysis of the data without requiring additional participant consent. All data analyzed in this study were deidentified. No financial compensation was provided to participants.

### Two-Finger Tapping Test and Feature Extraction

As part of the eFHS Offspring and Omni 1 protocol, participants were sent the 2-finger tapping test at enrollment and then every 2 months over the course of 1 year. The 2-finger tap task informed participants that the task measured tapping speed and instructed participants to put their phone on a flat surface to use 2 fingers to alternately tap the buttons on the screen while trying to time the taps to be as even as possible (Figure S1 in Multimedia Appendix 1, screenshot of task). Each test session lasted 20 seconds, during which participants alternated tapping on designated areas of the smartphone screen with 2 fingers of the left hand for 10 seconds, followed by 2 fingers of the right hand for 10 seconds, as quickly and consistently as possible. Data were included if the app recorded a valid 10-second trial with at least 1 tap event. We did not set additional performance-based exclusion thresholds, such as a minimum number of taps. Our goal was to capture the full range of tapping behavior, including slow, irregular, or instruction-nonadherent tapping, because these patterns may reflect cognitive function. A few examples of finger tapping results are shown in Figure S2 in Multimedia Appendix 1. A comprehensive set of features was extracted from the intertap interval (ITI) data to capture various aspects of motor performance. (1) Basic temporal features included the total number of taps per session as an indicator of effort, endurance, and overall speed, the mean ITI as measures of central tendency, and the SD of ITI as a measure of temporal variability. We also calculated the coefficient of variation of ITI to account for differences in overall tapping speed. (2) Asymmetry features included the alternating tap ratio (the proportion of left-right alternating taps), and the mean and SD of ITI for each finger. Skewness and kurtosis of the ITI distribution were also calculated to capture irregular tapping patterns. (3) Consistency metrics included ITI range. This feature reflected the stability of performance. (4) Fatigue and temporal drift were characterized using the ITI slope (the change in ITI over time during the trial) and the last-to-first ITI ratio. These features indicated whether tapping slowed or changed over the course of the session. (5) Microfluctuation feature included the Microfluctuation Index. This index was defined as the SD of successive ITI differences to capture subtle motor instability.

### Neuropsychological Assessment

The methodology for administering neuropsychological tests in FHS has been previously described [23]. Cognitive testing was first introduced in 1976, through brief cognitive screenings and expanded to a more comprehensive neuropsychological battery beginning in 1999 [13,24]. In the present study, we used 21 commonly administered neuropsychological tests to evaluate cognitive function across 4 domains (Table S1 in Multimedia Appendix 1) [25]. To address missing values of neuropsychological tests, we used multiple imputation by chained equations [26]. Distributions of the Hooper Visual Organization Test, Trail Making Test part A, and Trail Making Test part B were normalized using natural logarithmic transformation. To aid interpretation, the transformed scores were directionally adjusted so that higher values reflected better cognitive performance. All neuropsychological test scores were subsequently standardized to *z*-scores (mean 0, SD 1). These neuropsychological test scores were then summarized into 4 cognitive domains by averaging *z*-scores of neuropsychological tests within each domain: memory, executive function, language, and visuospatial function. A global cognitive score was further derived based on the mean values of all 4 cognitive domain scores.

### Statistical Analyses

Linear regression models were used to assess the associations between finger tapping performance and cognitive domain scores. For each participant, the mean value of each finger tapping feature across all available trials was calculated and used as the independent variable. Global cognitive function served as the primary dependent variable. All finger tapping features were standardized to *z*-scores to enable comparability across metrics. Models were adjusted for age at the time of the tapping assessment, sex, education, cohort (Offspring or Omni 1), handedness, and the time interval (in years) between the neuropsychological assessment and the baseline tapping test. As secondary analyses, we further explored associations with 4 specific cognitive domains: memory, executive function, language, and visuospatial function.

To further characterize hand-specific relationships, we performed stratified analyses for right- and left-hand tapping data. Sensitivity analyses were also conducted within the subset of participants identified as right-handed to reduce heterogeneity due to hand preference. Within this group, we reassessed the associations between each finger tapping metric and cognitive domain scores, additionally stratifying by the hand used during the tapping assessment (right vs left). In addition, we grouped the trials according to whether they were performed with the dominant or nondominant hand and repeated the association analyses within these groups. To explore the impact of noncompliant trials, we conducted a sensitivity analysis excluding trials (3729/4277, 12.8%) with an alternating tap ratio less than 0.80. To evaluate the potential impact of practice effects arising from different numbers of completed sessions, we performed a further sensitivity analysis to examine the associations between finger tapping measures and cognitive domain scores using each participant’s first 3 sessions. Bonferroni adjustment was used to correct multiple testing, and a significance threshold was determined with *P*<.05/15=.003, with the total number of finger tapping features. As an exploratory analysis, we examined whether cohort modified the association between finger tapping measures and cognition. In each linear regression model, we included an interaction term between the tapping feature and cohort.

We further used the least absolute shrinkage and selection operator (LASSO) regression to derive a composite score for global cognitive function based on finger tapping features. Specifically, we implemented a 5-fold cross-validation loop. For each training set, an inner 5-fold cross-validation was used to select the optimal regularization parameter (α). The composite score was defined as a weighted linear combination of the tapping features. The weights were the LASSO-derived coefficients within each training fold. The fitted model was then applied to the corresponding held-out test fold to obtain out-of-sample predicted global cognitive scores. Then, we assessed the association between the composite score and global cognitive function using partial correlation analysis adjusting for age, sex, and education. In addition, domain-specific composite tapping scores were constructed for each cognitive domain following the same procedure. All analyses were performed using *R* (version 4.0.3; R Core Team) or Python (version 3.6.8; Python Software Foundation).

## Results

### Cohort Descriptive

This study included 302 eFHS participants (mean age 74.7, SD 6.3 years; n=169, 56%, were female participants, n=192, 63.6%, had college degree or higher, and n=40, 13.2%, were non-White participants; Table 1). As expected, the majority (247/302, 81.8%) were right-handed, while 11.9% (36/302) were left-handed and 5% (15/302) were ambidextrous. On average, participants had completed 7 (SD 4) FTT sessions with both hands administered. Neurologically, the sample was generally healthy—the prevalence of PD was 0.3% (1/302). The average Mini-Mental State Exam (MMSE) score was 28.8 (SD 1.3), with 2.3% (7/302) of participants meeting criteria for MCI and 0.3% (1/302) for dementia. Self-rated health status was favorable—74.1% (224/302) of participants rated their general health as “very good” or “excellent.”

**Table 1.** Demographics of the study participants^a^.

Variable	Total (N=302)	Global cognitive performance		
		First tertile (n=101)	Second tertile (n=100)	Third tertile (n=101)
Age (y), mean (SD)	74.7 (6.3)	76.0 (7.0)	75.0 (6.0)	73.3 (5.5)
Sex, n (%)				
Females	169 (56)	52 (51.5)	52 (52)	65 (64.4)
Males	133 (44)	49 (48.5)	48 (48)	36 (35.6)
Omni cohort, n (%)	40 (13.2)	27 (26.7)	7 (7)	6 (5.9)
Education, n (%)				
High school	30 (9.9)	15 (14.9)	10 (10)	5 (5)
Some college	80 (26.5)	39 (38.6)	23 (23)	18 (17.8)
College and higher	192 (63.6)	47 (46.5)	67 (67)	78 (77.2)
Handedness, n (%)				
Right	247 (81.8)	81 (80.2)	84 (84)	82 (81.2)
Left	36 (11.9)	14 (13.9)	11 (11)	11 (10.9)
Ambidextrous	15 (5)	5 (5)	4 (4)	6 (5.9)
Unknown/missing	4 (1.3)	1 (1)	1 (1)	2 (2)
Time interval between the finger tapping task and the neuropsychological tests, mean (SD)	1.7 (1.2)	1.7 (1.2)	1.6 (1.1)	1.8 (1.3)
Number of finger tapping test session administered, mean (SD)	7 (4)	6 (4)	7 (4)	8 (3)
MMSE^b^, mean (SD)	28.8 (1.3)	28.1 (1.6)	29.0 (1.1)	29.4 (0.8)
Prevalent MCI^c^, n (%)	7 (2.3)	7 (6.9)	0	0
Prevalent dementia, n (%)	1 (0.3)	1 (1)	0	0
Prevalent Parkinson disease, n (%)	1 (0.3)	0	1 (1)	0
General health SF^d^-12, n (%)				
Poor	1 (0.3)	1 (1)	0	0
Fair	10 (3.3)	6 (5.9)	2 (2)	2 (2)
Good	67 (22.2)	26 (25.7)	25 (25)	16 (15.8)
Very good	146 (48.3)	45 (44.6)	47 (47)	54 (53.5)
Excellent	78 (25.8)	23 (22.8)	26 (26)	29 (28.7)

a Categorical variables are presented as counts (proportions). Continuous variables are presented as mean (SD).

b MMSE: Mini-Mental State Exam.

c MCI: mild cognitive impairment.

d SF: Short Form.

When stratified by tertiles of global cognitive function, participants in the third tertile (n=101) were younger (mean age 73.3 vs 76 years in the first tertile), more likely to be female participants (n=65, 64.4% vs n=52, 51.5%), and more highly educated (n=78, 77.2% with college or higher vs n=47, 46.5% in the first tertile). The proportion of participants from the Omni cohort (representing greater racial/ethnic diversity) was higher in the first tertile (n=27, 26.7%) compared to the highest (n=6, 5.9%). MMSE scores followed the expected gradient, increasing from 28.1 (SD 1.6) in the first tertile to 29.4 (SD 0.8) in the third tertile. Similarly, MCI and dementia cases were observed only in the first tertile group. Participants in the third tertile also completed more FTTs on average (17 vs 13). This suggested possible differences in adherence or engagement.

Participants who completed the digital finger tapping assessment were younger and more highly educated compared to those in the broader Offspring/Omni 1 cohort at Exam 10/5. Additional demographic comparisons with the full eFHS and Offspring/Omni 1 samples are presented in Table S2 in Multimedia Appendix 1. The distributions of finger tapping features and cognitive domain scores, including 25th percentile, median, and 75th percentile values, are shown in Tables S3 and S4**,** respectively, in Multimedia Appendix 1.

### Association Between Digital Finger Tapping Features and Cognitive Performance

Among the 15 finger tapping features evaluated, 11 were significantly associated with global cognitive function (*P* values<.001; Table 2). The strongest associations were observed for tapping variability metrics derived from ITI, such as SD, range, and Microfluctuation Index. For example, a 1 SD increase in mean ITI (average time differences between successive taps) was associated with a 0.23 decrease in global cognitive function score (*β*=−.23; 95% CI −0.30 to −0.17; *P*<.001). In addition to variability-based features, performance-related features such as number of taps (*β*=.14; 95% CI 0.07-0.21) were positively associated with global cognitive function.

**Table 2.** The association between digital finger tapping features and global cognitive function^a^.

Group and finger tapping features	Description	β (95% CI)	*P* value
Basic temporal features			
Number of taps	Indicator of effort, endurance, and speed	0.14 (0.07 to 0.21)	<.001
Mean ITI^b^	Average time differences between successive taps	–0.23 (–0.30 to –0.17)	<.001
SD of ITI	Measures tapping variability	–0.22 (–0.28 to –0.15)	<.001
CV^c^ of ITI	CV of ITI	–0.14 (–0.20 to –0.07)	<.001
Asymmetry features			
Skewness of ITI	Skewness of ITI	0.00 (–0.07 to 0.06)	.95
Kurtosis of ITI	Kurtosis of ITI	0.03 (–0.04 to 0.09)	.38
Alternating tap ratio	Fraction of alternating left-right taps	0.05 (–0.01 to 0.12)	.11
Mean of left ITI	Mean of tap times for the left finger	–0.22 (–0.29 to –0.16)	<.001
SD of left ITI	SD of tap times for the left finger	–0.18 (–0.25 to –0.11)	<.001
Mean of right ITI	Mean of tap times for the right finger	–0.20 (–0.27 to –0.14)	<.001
SD of right ITI	SD of tap times for the right finger	–0.12 (–0.19 to –0.05)	<.001
Consistency metrics			
ITI range	Difference between the longest and shortest ITI	–0.23 (–0.30 to –0.17)	<.001
Fatigue and temporal drift			
ITI slope	Change in ITI overtime	–0.11 (–0.17 to –0.05)	<.001
Last to first ITI	Ratio of last ITI to first ITI	–0.06 (–0.13 to 0.00)	.05
Microfluctuation feature			
Microfluctuation Index	SD of successive ITI differences	–0.16 (–0.22 to –0.09)	<.001

a Significant associations were claimed if *P*<.05/15=.0033. Features without laterality labels were computed using all taps across both buttons within each trial. Features labeled “left” or “right” were calculated separately for taps on the left and right buttons, respectively.

b ITI: intertap interval.

c CV: coefficient of variation.

Beyond global cognitive function, we further examined the associations between finger tapping features and 4 domain-specific cognitive outcomes: memory, executive function, language, and visuospatial function (Table 3, Table S5 in Multimedia Appendix 1). Similar to findings for global cognition, variability in ITI emerged as a consistent predictor across domains. Executive function demonstrated the strongest associations, with 11 out of 15 finger tapping features reaching significance. The most significant association was observed for mean ITI (*β*=−0.27; 95% CI −0.33 to −0.20; *P*<.001). Visuospatial function showed fewer associations (6 significant features), yet ITI range again emerged as the strongest predictor (*β*=−0.26; 95% CI −0.38 to −0.15; *P*<.001). When we applied a false discovery rate correction across all 75 finger tapping-cognition tests, the overall pattern remained similar. Ten features remained significant for memory, 11 for executive function, 10 for language, 10 for visuospatial function, and 11 for global cognitive function (all with false discovery rate <.05). After excluding trials with an alternating tap ratio less than 0.8, the pattern of the associations between tapping metrics and cognitive domains remained similar (Table S6 in Multimedia Appendix 1). In additional sensitivity analysis, we used the first 3 sessions of each participant. The directions of the associations between finger tapping measures and cognitive domains were largely consistent, but fewer associations were statistically significant (Table S7 in Multimedia Appendix 1). Across all domains, 10 (13.3%) interactions reached nominal significance (Table S8 in Multimedia Appendix 1). For global cognitive function, the most significant interaction was for ITI slope (*P*=.03). We conducted a sensitivity analysis using complete-case data by excluding participants with missing neuropsychological test scores (Table S9 in Multimedia Appendix 1). Most associations remained significant with the same direction of effects.

**Table 3.** The most significant digital finger tapping feature for each cognitive domain^a^.

Cognitive domain	Number of significant finger tapping features, n	Most significant association		
		Finger tapping features	β (95% CI)	*P* value
Memory	9	ITI^b^ range	–0.19 (–0.26 to –0.12)	<.001
Executive function	11	Mean ITI	–0.27 (–0.33 to –0.20)	<.001
Language	9	Mean of right ITI	–0.24 (–0.32 to –0.16)	<.001
Visuospatial function	6	ITI Range	–0.26 (–0.38 to –0.15)	<.001

a Significant associations were claimed if *P*<.05/15=.003.

b ITI: intertap interval.

Given the presence of multiple finger tapping features associated with cognitive performance, we constructed a composite tapping score for each cognitive test domain using LASSO regression. The finger tapping composite scores were significantly correlated with cognitive domain scores (all with *P*<.001). The strength of these correlations differed by domain. Partial correlation coefficients were 0.21 for memory, 0.22 for executive function, 0.38 for language, 0.23 for visuospatial function, and 0.41 for global cognition.

We further examined the associations between finger tapping features and cognitive function by analyzing FTTs administered separately to the left and right hands (Tables S5, S10, and S11 in Multimedia Appendix 1). Overall, the direction and significance of associations observed in the combined-hand models were largely consistent with those identified in hand-specific analyses, supporting the robustness of the findings across tapping conditions. For global cognitive function, 9 features remained significantly associated in both left-hand and right-hand models. For example, 2 key features of temporal control, including the mean ITI and ITI range, were consistently associated with lower global cognition across all 3 models. Across domain-specific outcomes, effect directions were again broadly consistent. For executive function, the number of significant tapping features was similar across hands (10 in left-hand and 8 in right-hand models), with mean ITI or mean right-hand ITI consistently emerging as the most predictive features. Language and memory domains also demonstrated robust associations, particularly with right-hand tapping metrics such as ITI range and mean ITI. In contrast, associations for visuospatial function were more pronounced in the right-hand model (6 significant features) than the left-hand model (1 significant feature). This asymmetry may reflect functional lateralization of spatial processing or task engagement differences between hands. Additionally, the predominance of right-handed individuals in the sample (247/302, 81.8%) may have contributed to the stronger associations observed in right-hand tapping performance.

To further examine the robustness of associations and minimize potential confounding from handedness, we conducted subgroup analyses restricted to right-handed participants (n=247; Tables S12-S14 in Multimedia Appendix 1). While differences in the number of significant tapping features emerged across cognitive domains, the direction of effects, particularly for temporal variability features, remained consistent. When we reanalyzed the data by dominant versus nondominant hand, the overall pattern of associations was similar (Tables S15 and S16 in Multimedia Appendix 1). For executive function, however, more finger tapping measures were significantly associated with performance when tests were administered with the nondominant hand (n=10) than with the dominant hand (n=8). In dominant-hand trials, mean ITI showed the most significant associations across all cognitive domains. In nondominant hand trials, mean ITI again showed the strongest associations with memory, executive function, and language. However, the ITI range, reflecting temporal variability, was most significantly associated with visuospatial and global cognitive function.

## Discussion

### Principal Results

In this study of predominantly cognitively intact, community-based older adults, several digital finger tapping features were significantly associated with cognitive performance. These results support the use of motor-based digital measures as a tool for evaluating cognitive health. Notably, variability features such as the SD, coefficient of variation, ITI range, and the Microfluctuation Index were consistently associated with cognitive domains. These results suggest that temporal features derived from simple tapping tasks may serve as sensitive and scalable indicators of general cognitive functioning.

Executive function emerged as the most consistently associated domain, showing strong relationships with 13 finger tapping features. Given that executive function is a multidimensional construct encompassing key cognitive processes such as processing speed, planning, working memory, and inhibitory control [27], this finding provides important insight into the potential cognitive mechanisms underlying fine motor performance. Although the finger tapping task appears simple, its successful execution in a timed context requires the ability to sustain and update task goals (working memory) [28], initiate and maintain a consistent motor rhythm (planning and motor initiation) [29], and suppress irrelevant motor impulses (inhibitory control) [30]. The strong associations between temporal tapping features, particularly variability-based features, and executive function suggest that digital motor timing tasks may serve as sensitive proxies for detecting early executive dysfunction. Interestingly, language performance was most strongly associated with mean tap times for the right finger of both right and left hands. This suggested that beyond just timing or variability, the dynamics of motor execution may have a specific connection with language processing abilities. This finding is consistent with prior research indicating shared neural substrates for motor and language functions [31]. And it provides behavioral evidence for the functional interdependence between these domains.

Stratified analyses by hand and by handedness revealed generally consistent associations between finger tapping features and cognitive performance. Across both left- and right-hand administered tests, key temporal metrics such as SD of ITI, ITI range, and Microfluctuation Index remained associated with global cognitive function. However, subtle differences emerged in the number and strength of significant associations across hands and cognitive domains. For instance, visuospatial associations were more prominent in right-hand tests, while left-hand results showed fewer significant features overall. Analyses restricted to right-handed participants largely confirmed the main findings, with core tapping features such as mean ITI and ITI range consistently emerging as the informative predictors across domains. These results reinforce the generalizability of tapping-derived digital biomarkers, while also suggesting that motor laterality and hand-specific administration may modestly influence the association. While many tapping features showed associations with cognition, 4 features, including skewness and kurtosis of ITI, alternating tap ratio, and last-to-first ITI ratio, were not significantly related to any cognitive domain. The alternating tap ratio is primarily an index of adherence to the alternating-tap instruction. However, maintaining an alternating pattern relies on attention and executive function. Therefore, variation in this measure may carry cognitively relevant information rather than simply indicating noncompliance to following instructions. For this reason, we included it in our analyses, although we did not observe significant associations with any cognitive domain. These findings highlight the need to prioritize the most informative digital biomarkers when evaluating cognitive health. Although these associations reached statistical significance, the effect sizes were small. This suggests that finger tapping measures explain only a modest proportion of variation in cognitive performance. From a clinical standpoint, these findings also underscore the growing relevance of digital phenotyping as a complementary approach to traditional cognitive screening [32]. Early signs of executive dysfunction and cognitive decline are often subtle and can be overlooked, especially in high-functioning or highly educated individuals. The associations identified between fine motor irregularities and cognitive performance suggest that brief, app-based tapping tasks may serve as passive indicators of early neurocognitive vulnerability.

This study offers several notable strengths. First, it leverages a comprehensive battery of neuropsychological assessments covering four major cognitive domains, enabling robust evaluation of cognitive function. Second, the repeated administration of digital FTTs at regular intervals provides a more detailed characterization of motor performance over time and its association with cognitive outcomes. While digital finger tapping tasks have been explored in prior studies, they have predominantly focused on targeted clinical populations, such as individuals with PD or other neurodegenerative disorders [10,33,34]. In contrast, our study is among the first to investigate smartphone-based finger tapping performance in a large, community-based sample of older adults. The majority of participants were subjectively healthy and free of diagnosed cognitive impairment. This distinction enhances the generalizability of our findings and supports the broader application of digital motor assessments for population-level cognitive monitoring. The use of a smartphone-based tapping task, coupled with the derivation of multiple temporal and variability-based digital metrics, represents a novel, noninvasive, and scalable approach for assessing fine motor control in real-world settings. Notably, a high proportion of older iPhone users completed and returned the tapping assessments. Many participants completed multiple administrations. Given the well-documented challenges of adherence in digital health studies, this level of engagement suggests that the task was both accessible and acceptable to participants. Additionally, stratification of tapping performance by left and right hand allows for the exploration of potential lateralization effects. This offers further insight into the relationship between motor function and cognitive domains.

### Comparison With Prior Work

Our findings align with prior research using the traditional method of assessing finger tapping speed [35]. In the earlier study, finger tapping was featured by instructing participants to repeatedly press a mechanical lever with an attached counter using the index finger of each hand over a fixed 10-second interval. It demonstrated that individuals who later converted to MCI exhibited a faster decline in tapping speed compared to nonconverters. Similarly, in our study, a lower number of taps (slower tapping speed) was significantly associated with lower global and domain-specific cognitive performance, supporting motor slowing as a behavioral marker of cognition.

### Limitations

First, this study was cross-sectional and conducted in a sample consisting predominantly of cognitively intact older adults. Although finger tapping features were collected longitudinally, the cognitive assessments were not temporally aligned with each tapping session. Therefore, we summarized finger tapping performance by averaging repeated trials into a single mean value per participant. This approach allowed us to focus on between-person differences in mean tapping performance but did not capture within-person variability or change over time. In addition, the time interval between neuropsychological test and finger tapping (mean 1.7, SD 1.2 y) limited inferences about how well tapping performance reflects real-time cognitive status. As longitudinal follow-up data become available, future work will evaluate whether tapping metrics can predict subsequent cognitive decline or discriminate between cognitively impaired and unimpaired individuals.

Second, the first step of the 2-finger tap app entitled tapping speed informed the participant that “this activity measures your tapping speed.” However, the instructions for the left hand and right hand in steps 2 and 4 include the statement “try to make your taps as even as possible” (Figure S1 in Multimedia Appendix 1). These instructions were designed to encourage fast tapping with a stable rhythm. In practice, this may have led some participants to focus more on speed and others more on regularity. In this context, ITI variability may reflect both intrinsic motor control and individual task strategies. Our findings should be interpreted accordingly. Future studies could use separate speed-focused and regularity-focused conditions to better disentangle these components as both may reflect early neurodegenerative processes. Besides, we examined only the alternating 2-finger version of the FTT with a fixed 10-second trial duration. Because of this short duration, the ITI slope may capture rhythm establishment rather than physiological fatigue. It should be interpreted with caution. Future work should compare alternating and single-finger tapping protocols and systematically vary test duration to better separate rhythm establishment from physiological fatigue. This also can help determine the optimal design for digital tapping-based cognitive assessment.

Third, while the smartphone-based tapping task provides a standardized and practical assessment platform, performance may still be influenced by external factors such as participants’ familiarity with digital devices or technical variability in smartphone hardware and software. Although this study adjusted for age, sex, education, and handedness, conditions such as arthritis, motor fatigue, medication use, or depression may independently affect tapping speed, rhythm, or consistency. In some cases, motor slowing may reflect physical rather than cognitive decline. Therefore, these factors should be considered when interpreting tapping metrics in clinical practice, especially in people with multiple comorbidities.

Finally, the tapping task was only available on iOS devices. Therefore, 133 Android users were excluded. Because iOS and Android ownership differs by socioeconomic and demographic factors, this restriction may introduce socioeconomic selection bias. In addition, participants in our sample were generally well-educated and reported good to excellent subjective health. These characteristics may limit the generalizability of our findings to more diverse populations with different demographic or clinical profiles. Moreover, cognitive performance was high in this cohort (mean MMSE 28.8, SD 1.3; dementia prevalence 0.3%), suggesting possible ceiling effects in the cognitive measures. Such ceiling effects may attenuate observed associations and underestimate the true relationships that may be more apparent in clinically diverse populations. In addition, the iPhone models and variation in touchscreen sampling rates or input latency across devices may introduce measurement noise into fine-grained variability metrics (eg, SD of ITI and Microfluctuation Index). Further research in larger and more diverse populations with standardized hardware is needed to confirm these findings.

### Conclusions

In summary, this study identified several finger tapping features that were significantly associated with performance across multiple cognitive domains and highlighted the potential of digital finger tapping tasks as informative biomarkers for assessing cognitive function. These findings suggest that finger tapping tasks may be used as noninvasive, scalable tools to assess cognitive performance.

## Supplementary material

10.2196/82463 Multimedia Appendix 1 Supplemental tables and figures.

## Abbreviations

eFHS: electronic Framingham Heart Study
FHS: Framingham Heart Study
FTT: finger tapping test
ITI: intertap interval
LASSO: least absolute shrinkage and selection operator
MCI: mild cognitive impairment
MMSE: Mini-Mental State Exam
PD: Parkinson disease
