# Supplementary material for: Associations Between Smartphone-Based Finger Tapping and Cognitive Performance in Older Adults: Observational Study
Source: J Med Internet Res. 2026 Feb 18;28:e82463. doi: 10.2196/82463 (PMC12916091; doi:10.2196/82463)
Supplement: Multimedia Appendix 1 [file jmir-v28-e82463-s001.docx]

**Supplemental Table 1.** Cognitive domains, corresponding NP tests, and missing data rates

| **Cognitive domain** | **NP test** | **Missing rate (%)** |
| --- | --- | --- |
| Memory | WMS Logical Memory (LM) Story A-Immediate Recall  LM - Delayed Recall  LM - Delayed Recognition  WMS Visual Reproductions (VR) - Immediate Recall  VR - Delayed Recall  VR - Delayed Recognition  WMS (Paired) Associate Learning (PA) - Immediate Recall  PA - Delayed Recall  PA - Delayed Recognition | 2.32  2.32  3.31  1.32  1.66  2.98  5.96  5.96  6.95 |
| Executive function | Trail Making Test A (Trails A)  Test B (Trails B)  WAIS - Digit Span Forward  Backward  WAIS - Similarities  WAIS-IV - Coding | 14.24  18.21  0.66  5.30  1.66  6.95 |
| Language | Controlled Oral Word Association Test (FAS)  Category Fluency (Animals)  Boston Naming Test – 36-item version  Boston Naming Test – 30-item version  Boston Naming Test – 10-item version | 4.30  4.30  9.93  9.93  9.93 |
| Visuospatial function | Hooper Visual Organization Test | 3.97 |

Abbreviations: WMS, Wechsler Memory Scale; WAIS, Wechsler Adult Intelligence Scale.

**Supplemental Table 2**. Comparison of demographics among group 1 (study participants), group 2 (all eFHS participants), and group 3 (all Offspring and Omni 1 participants at Exam 10 and Exam 5).

| **Variable** | **Group 1**  **(n=302)** | **Group 2**  **(n=620)** | **Group 3**  **(n= 1697)** |
| --- | --- | --- | --- |
| **Age (years)** | 74.7 (6.3) | 73.6 (6.3) | 76.4 (7.7) |
| **Sex** |  |  |  |
| Women | 169 (56.0%) | 353 (56.9%) | 963 (56.7%) |
| Men | 133 (44.0%) | 267 (43.1%) | 734 (43.3%) |
| **Omni cohort** | 40 (13.2%) | 88 (14.2%) | 197 (11.6%) |
| **Education** |  |  |  |
| High school did not graduate | 0 | 3 (0.5%) | 36 (2.1%) |
| High school | 30 (9.9%) | 68 (11.0%) | 337 (19.9%) |
| Some college | 80 (26.5%) | 161 (26.0%) | 455 (26.8%) |
| College and higher | 192 (63.6%) | 384 (61.9%) | 843 (49.7%) |
| **Handedness** |  |  |  |
| Right | 247 (81.8%) | 510 (82.3%) | 1313 (77.4%) |
| Left | 36 (11.9%) | 63 (10.2%) | 158 (9.3%) |
| Ambidextrous | 15 (5.0%) | 17 (2.7%) | 67 (3.9%) |
| Unknown/missing | 4 (1.3%) |  | 159 (9.4%) |
| **MMSE** | 28.8 (1.3) | 28.7 (1.5) | 28.1 (2.3) |
| **Prevalent MCI** | 7 (2.3%) | 16 (2.6%) | 91 (5.4%) |
| **Prevalent dementia** | 1 (0.3%) | 1 (0.2%) | 28 (1.7%) |
| **Prevalent Parkinson’s disease** | 1 (0.3%) | 2 (0.3%) | 17 (1.0%) |
| **General health SF-12** |  |  |  |
| Poor | 1 (0.3%) | 2 (0.3%) | 15 (0.9%) |
| Fair | 10 (3.3%) | 23 (3.7%) | 95 (5.6%) |
| Good | 67 (22.2%) | 158 (25.5%) | 496 (29.2%) |
| Very good | 146 (48.3%) | 291 (46.9%) | 737 (43.4%) |
| Excellent | 78 (25.8%) | 144 (23.2%) | 335 (19.7%) |

Note: Categorical variables are presented as counts (%), and continuous variables are presented as mean (SD).

**Supplemental Table 3**. Distribution of finger tapping features

| **Group** | **Features** | **Total (n=302)** | | | **Global cognitive performance** | | | | | | | | |
| --- | --- | --- | --- | --- | --- | --- | --- | --- | --- | --- | --- | --- | --- |
|  |  |  |  |  | **First tertile (n=101)** | | | **Second tertile (n=100)** | | | **Third tertile (n=101)** | | |
|  |  | **25%** | **50%** | **75%** | **25%** | **50%** | **75%** | **25%** | **50%** | **75%** | **25%** | **50%** | **75%** |
| Basic temporal features | Number of taps | 27.8125 | 39.9375 | 58.1136 | 23.6154 | 31.0000 | 48.7500 | 29.7115 | 42.1250 | 55.6531 | 36.5833 | 49.6000 | 68.6818 |
|  | Mean ITI | 0.1939 | 0.2844 | 0.3990 | 0.2675 | 0.3581 | 0.5109 | 0.2032 | 0.2702 | 0.3494 | 0.1553 | 0.2202 | 0.2966 |
|  | SD of ITI | 0.0567 | 0.0890 | 0.1400 | 0.0819 | 0.1220 | 0.2349 | 0.0549 | 0.0928 | 0.1315 | 0.0497 | 0.0661 | 0.0895 |
|  | CV of ITI | 0.2638 | 0.3519 | 0.4445 | 0.2897 | 0.3689 | 0.5336 | 0.2672 | 0.3522 | 0.4389 | 0.2390 | 0.3251 | 0.4025 |
| Asymmetry features | Skewness of ITI | 0.6753 | 1.0817 | 1.4992 | 0.7590 | 1.1496 | 1.6126 | 0.7785 | 1.1533 | 1.3905 | 0.6370 | 0.9572 | 1.4574 |
|  | Kurtosis of ITI | 5.0348 | 6.6829 | 8.3877 | 5.0199 | 6.8498 | 8.1619 | 4.9857 | 6.7094 | 8.4096 | 5.2272 | 6.3534 | 8.4393 |
|  | Alternating Tap Ratio | 0.8773 | 0.9439 | 0.9759 | 0.8704 | 0.9357 | 0.9697 | 0.8748 | 0.9333 | 0.9737 | 0.8981 | 0.9612 | 0.9817 |
|  | Mean of Left ITI | 0.3889 | 0.5602 | 0.7843 | 0.4696 | 0.7189 | 0.9907 | 0.4037 | 0.5607 | 0.7032 | 0.3174 | 0.4467 | 0.6069 |
|  | SD of Left ITI | 0.0850 | 0.1407 | 0.2352 | 0.1183 | 0.2080 | 0.3452 | 0.0930 | 0.1421 | 0.2290 | 0.0703 | 0.1066 | 0.1485 |
|  | Mean of Right ITI | 0.3786 | 0.5667 | 0.7724 | 0.4970 | 0.6825 | 1.0251 | 0.4048 | 0.5745 | 0.7094 | 0.3076 | 0.4424 | 0.5870 |
|  | SD of Right of ITI | 0.0794 | 0.1324 | 0.2126 | 0.1078 | 0.1587 | 0.3116 | 0.0826 | 0.1376 | 0.2267 | 0.0633 | 0.0998 | 0.1467 |
| Consistency metrics | ITI Range | 0.2983 | 0.4255 | 0.6248 | 0.3872 | 0.5688 | 0.9567 | 0.3011 | 0.4321 | 0.6168 | 0.2523 | 0.3316 | 0.4544 |
| Fatigue and temporal drift | ITI Slope | -0.0043 | -0.0012 | -0.0001 | -0.0115 | -0.0021 | -0.0004 | -0.0042 | -0.0012 | -0.0002 | -0.0015 | -0.0006 | 0.0001 |
|  | Last to First ITI | 0.7492 | 0.9080 | 1.1191 | 0.7679 | 0.8967 | 1.3745 | 0.7430 | 0.9170 | 1.1430 | 0.7463 | 0.9034 | 1.0478 |
| Micro fluctuation feature | Micro Fluctuation Index | 0.0773 | 0.1190 | 0.1943 | 0.1146 | 0.1594 | 0.3298 | 0.0758 | 0.1176 | 0.1638 | 0.0680 | 0.0942 | 0.1270 |

**Supplemental Table 4**. The distribution of cognitive domain scores

| **Cognitive domain** | **25%** | **50%** | **75%** |
| --- | --- | --- | --- |
| Memory | -0.42 | 0.08 | 0.49 |
| Executive function | -0.39 | 0.03 | 0.45 |
| Language | -0.37 | 0.16 | 0.50 |
| Visuospatial function | -0.41 | 0.14 | 0.65 |
| Global cognitive function | -0.30 | 0.12 | 0.44 |

**Supplemental Table 5**. The association between digital finger tapping features and cognitive domains

| **Cognitive domain** | **Finger tapping feature** | **Both hands** | | | **Left-hand administrated** | | | **Right-hand administrated** | | |
| --- | --- | --- | --- | --- | --- | --- | --- | --- | --- | --- |
|  |  | **β** | **95% CI** | ***P* value** | **β** | **95% CI** | ***P* value** | **β** | **95% CI** | ***P* value** |
| Memory | Number of taps | 0.11 | 0.04, 0.19 | 4.11E-03 | 0.12 | 0.04, 0.19 | 3.02E-03 | 0.09 | 0.01, 0.16 | 2.72E-02 |
|  | Mean ITI | -0.18 | -0.26, -0.11 | 1.09E-06 | -0.14 | -0.21, -0.07 | 2.23E-04 | -0.12 | -0.19, -0.04 | 2.27E-03 |
|  | SD of ITI | -0.18 | -0.25, -0.10 | 2.93E-06 | -0.10 | -0.18, -0.03 | 7.50E-03 | -0.14 | -0.21, -0.07 | 1.63E-04 |
|  | CV of ITI | -0.13 | -0.21, -0.06 | 3.38E-04 | -0.08 | -0.15, -0.01 | 3.31E-02 | -0.13 | -0.20, -0.06 | 4.50E-04 |
|  | Skewness of ITI | -0.03 | -0.11, 0.04 | 3.67E-01 | -0.02 | -0.10, 0.05 | 5.08E-01 | -0.04 | -0.11, 0.03 | 2.72E-01 |
|  | Kurtosis of ITI | 0.00 | -0.07, 0.07 | 9.57E-01 | 0.00 | -0.07, 0.08 | 9.28E-01 | 0.01 | -0.07, 0.08 | 8.60E-01 |
|  | Alternating Tap Ratio | 0.06 | -0.01, 0.14 | 8.65E-02 | 0.06 | -0.01, 0.13 | 1.05E-01 | 0.05 | -0.02, 0.12 | 1.79E-01 |
|  | Mean of Left ITI | -0.18 | -0.25, -0.10 | 3.44E-06 | -0.13 | -0.20, -0.05 | 9.38E-04 | -0.13 | -0.20, -0.05 | 7.57E-04 |
|  | SD of Left ITI | -0.14 | -0.22, -0.07 | 2.13E-04 | -0.08 | -0.15, -0.00 | 4.86E-02 | -0.15 | -0.22, -0.08 | 3.55E-05 |
|  | Mean of Right ITI | -0.17 | -0.24, -0.09 | 1.52E-05 | -0.15 | -0.22, -0.07 | 9.82E-05 | -0.12 | -0.20, -0.04 | 1.87E-03 |
|  | SD of Right ITI | -0.13 | -0.20, -0.05 | 1.05E-03 | -0.10 | -0.17, -0.02 | 1.30E-02 | -0.12 | -0.19, -0.05 | 1.33E-03 |
|  | ITI Range | -0.19 | -0.26, -0.12 | 6.28E-07 | -0.11 | -0.19, -0.04 | 2.86E-03 | -0.16 | -0.23, -0.09 | 1.93E-05 |
|  | ITI Slope | -0.07 | -0.14, -0.00 | 4.62E-02 | 0.06 | -0.02, 0.13 | 1.29E-01 | 0.02 | -0.05, 0.09 | 6.09E-01 |
|  | Last to First ITI | -0.03 | -0.10, 0.04 | 4.56E-01 | -0.03 | -0.11, 0.04 | 3.58E-01 | 0.01 | -0.06, 0.09 | 6.92E-01 |
|  | Micro Fluctuation Index | -0.14 | -0.21, -0.06 | 2.93E-04 | -0.10 | -0.17, -0.03 | 6.49E-03 | -0.13 | -0.20, -0.06 | 3.75E-04 |
| Executive function | Number of taps | 0.16 | 0.09, 0.23 | 2.16E-05 | 0.15 | 0.08, 0.23 | 4.21E-05 | 0.13 | 0.06, 0.20 | 1.90E-04 |
|  | Mean ITI | -0.27 | -0.33, -0.20 | 2.64E-14 | -0.19 | -0.26, -0.12 | 1.70E-07 | -0.18 | -0.25, -0.12 | 2.02E-07 |
|  | SD of ITI | -0.24 | -0.31, -0.17 | 9.82E-12 | -0.15 | -0.22, -0.08 | 2.29E-05 | -0.15 | -0.21, -0.08 | 1.94E-05 |
|  | CV of ITI | -0.12 | -0.19, -0.05 | 1.23E-03 | -0.11 | -0.18, -0.04 | 1.80E-03 | -0.08 | -0.14, -0.01 | 2.73E-02 |
|  | Skewness of ITI | 0.03 | -0.04, 0.10 | 4.05E-01 | 0.00 | -0.07, 0.07 | 9.45E-01 | 0.01 | -0.06, 0.08 | 7.55E-01 |
|  | Kurtosis of ITI | 0.06 | -0.01, 0.12 | 1.11E-01 | 0.03 | -0.04, 0.10 | 4.46E-01 | 0.06 | -0.01, 0.12 | 1.03E-01 |
|  | Alternating Tap Ratio | 0.05 | -0.02, 0.12 | 1.38E-01 | 0.08 | 0.01, 0.15 | 2.38E-02 | 0.03 | -0.04, 0.10 | 4.20E-01 |
|  | Mean of Left ITI | -0.26 | -0.33, -0.19 | 3.06E-13 | -0.17 | -0.24, -0.10 | 1.83E-06 | -0.18 | -0.25, -0.11 | 3.83E-07 |
|  | SD of Left ITI | -0.18 | -0.25, -0.11 | 1.03E-06 | -0.14 | -0.21, -0.07 | 1.75E-04 | -0.16 | -0.22, -0.09 | 3.39E-06 |
|  | Mean of Right ITI | -0.21 | -0.28, -0.14 | 1.44E-08 | -0.19 | -0.26, -0.12 | 9.19E-08 | -0.17 | -0.24, -0.11 | 1.00E-06 |
|  | SD of Right ITI | -0.12 | -0.20, -0.05 | 7.96E-04 | -0.12 | -0.20, -0.05 | 7.74E-04 | -0.09 | -0.16, -0.02 | 1.04E-02 |
|  | ITI Range | -0.25 | -0.31, -0.18 | 6.64E-12 | -0.17 | -0.24, -0.10 | 2.02E-06 | -0.16 | -0.23, -0.09 | 4.17E-06 |
|  | ITI Slope | -0.15 | -0.22, -0.09 | 9.13E-06 | 0.05 | -0.02, 0.12 | 1.89E-01 | 0.00 | -0.07, 0.06 | 8.90E-01 |
|  | Last to First ITI | -0.05 | -0.12, 0.02 | 1.53E-01 | -0.04 | -0.11, 0.03 | 2.74E-01 | 0.00 | -0.07, 0.06 | 9.23E-01 |
|  | Micro Fluctuation Index | -0.16 | -0.23, -0.09 | 4.55E-06 | -0.16 | -0.23, -0.09 | 1.14E-05 | -0.14 | -0.21, -0.07 | 5.00E-05 |
| Language | Number of taps | 0.17 | 0.08, 0.25 | 1.16E-04 | 0.15 | 0.07, 0.24 | 3.51E-04 | 0.15 | 0.07, 0.24 | 3.68E-04 |
|  | Mean ITI | -0.22 | -0.30, -0.14 | 1.40E-07 | -0.16 | -0.24, -0.08 | 9.34E-05 | -0.21 | -0.29, -0.13 | 4.42E-07 |
|  | SD of ITI | -0.21 | -0.29, -0.13 | 4.89E-07 | -0.14 | -0.22, -0.06 | 6.75E-04 | -0.19 | -0.27, -0.11 | 3.41E-06 |
|  | CV of ITI | -0.13 | -0.21, -0.05 | 1.24E-03 | -0.09 | -0.17, -0.01 | 3.43E-02 | -0.11 | -0.19, -0.03 | 5.83E-03 |
|  | Skewness of ITI | -0.04 | -0.12, 0.04 | 3.56E-01 | -0.01 | -0.09, 0.07 | 8.49E-01 | -0.06 | -0.14, 0.02 | 1.38E-01 |
|  | Kurtosis of ITI | 0.00 | -0.08, 0.08 | 9.48E-01 | 0.03 | -0.05, 0.11 | 4.51E-01 | -0.02 | -0.10, 0.06 | 6.46E-01 |
|  | Alternating Tap Ratio | 0.02 | -0.06, 0.10 | 6.38E-01 | 0.03 | -0.05, 0.11 | 4.09E-01 | -0.01 | -0.09, 0.07 | 8.04E-01 |
|  | Mean of Left ITI | -0.22 | -0.30, -0.14 | 1.92E-07 | -0.17 | -0.25, -0.09 | 5.07E-05 | -0.20 | -0.28, -0.12 | 1.73E-06 |
|  | SD of Left ITI | -0.21 | -0.29, -0.13 | 7.48E-07 | -0.18 | -0.26, -0.10 | 1.46E-05 | -0.12 | -0.20, -0.04 | 2.69E-03 |
|  | Mean of Right ITI | -0.24 | -0.32, -0.16 | 6.32E-09 | -0.19 | -0.27, -0.11 | 3.75E-06 | -0.22 | -0.30, -0.13 | 2.85E-07 |
|  | SD of Right ITI | -0.11 | -0.19, -0.02 | 1.16E-02 | -0.07 | -0.15, 0.01 | 9.60E-02 | -0.11 | -0.19, -0.03 | 7.14E-03 |
|  | ITI Range | -0.24 | -0.32, -0.16 | 1.42E-08 | -0.17 | -0.25, -0.09 | 5.63E-05 | -0.20 | -0.28, -0.12 | 1.26E-06 |
|  | ITI Slope | -0.06 | -0.14, 0.02 | 1.13E-01 | 0.05 | -0.03, 0.13 | 2.56E-01 | -0.04 | -0.12, 0.04 | 3.21E-01 |
|  | Last to First ITI | -0.05 | -0.13, 0.03 | 1.91E-01 | -0.06 | -0.14, 0.02 | 1.40E-01 | 0.00 | -0.08, 0.08 | 9.68E-01 |
|  | Micro Fluctuation Index | -0.18 | -0.26, -0.10 | 1.53E-05 | -0.14 | -0.22, -0.06 | 5.92E-04 | -0.15 | -0.23, -0.07 | 2.22E-04 |
| Visuospatial | Number of taps | 0.11 | -0.01, 0.23 | 7.38E-02 | 0.09 | -0.03, 0.21 | 1.44E-01 | 0.10 | -0.02, 0.22 | 9.35E-02 |
|  | Mean ITI | -0.26 | -0.37, -0.14 | 1.60E-05 | -0.15 | -0.27, -0.04 | 9.95E-03 | -0.23 | -0.35, -0.11 | 1.16E-04 |
|  | SD of ITI | -0.25 | -0.36, -0.13 | 2.38E-05 | -0.13 | -0.25, -0.02 | 2.41E-02 | -0.23 | -0.34, -0.11 | 9.38E-05 |
|  | CV of ITI | -0.17 | -0.28, -0.06 | 3.38E-03 | -0.15 | -0.26, -0.03 | 1.26E-02 | -0.14 | -0.25, -0.03 | 1.27E-02 |
|  | Skewness of ITI | 0.03 | -0.08, 0.15 | 5.72E-01 | 0.03 | -0.08, 0.15 | 5.74E-01 | -0.01 | -0.12, 0.11 | 9.19E-01 |
|  | Kurtosis of ITI | 0.06 | -0.06, 0.17 | 3.31E-01 | 0.02 | -0.09, 0.14 | 6.92E-01 | 0.07 | -0.04, 0.18 | 2.40E-01 |
|  | Alternating Tap Ratio | 0.08 | -0.03, 0.19 | 1.59E-01 | 0.09 | -0.03, 0.20 | 1.41E-01 | 0.08 | -0.03, 0.20 | 1.40E-01 |
|  | Mean of Left ITI | -0.25 | -0.36, -0.13 | 3.28E-05 | -0.14 | -0.26, -0.02 | 2.29E-02 | -0.21 | -0.33, -0.10 | 3.80E-04 |
|  | SD of Left ITI | -0.20 | -0.31, -0.08 | 1.05E-03 | -0.14 | -0.26, -0.03 | 1.70E-02 | -0.20 | -0.32, -0.09 | 3.55E-04 |
|  | Mean of Right ITI | -0.20 | -0.32, -0.09 | 6.38E-04 | -0.17 | -0.29, -0.06 | 3.28E-03 | -0.21 | -0.33, -0.10 | 4.01E-04 |
|  | SD of Right ITI | -0.12 | -0.24, -0.00 | 4.48E-02 | -0.09 | -0.21, 0.03 | 1.37E-01 | -0.13 | -0.25, -0.02 | 2.14E-02 |
|  | ITI Range | -0.26 | -0.38, -0.15 | 1.20E-05 | -0.17 | -0.29, -0.05 | 4.31E-03 | -0.22 | -0.33, -0.10 | 2.01E-04 |
|  | ITI Slope | -0.15 | -0.26, -0.04 | 6.44E-03 | 0.08 | -0.04, 0.20 | 1.79E-01 | -0.14 | -0.24, -0.03 | 1.52E-02 |
|  | Last to First ITI | -0.12 | -0.23, -0.01 | 2.70E-02 | -0.07 | -0.18, 0.04 | 2.36E-01 | -0.10 | -0.20, 0.01 | 8.97E-02 |
|  | Micro Fluctuation Index | -0.15 | -0.27, -0.04 | 8.29E-03 | -0.13 | -0.25, -0.02 | 2.57E-02 | -0.17 | -0.28, -0.05 | 4.15E-03 |
| Global cognitive function | Number of taps | 0.14 | 0.07, 0.21 | 1.15E-04 | 0.13 | 0.06, 0.20 | 3.10E-04 | 0.12 | 0.05, 0.19 | 6.27E-04 |
|  | Mean ITI | -0.23 | -0.30, -0.17 | 5.95E-12 | -0.16 | -0.23, -0.10 | 2.53E-06 | -0.19 | -0.25, -0.12 | 4.29E-08 |
|  | SD of ITI | -0.22 | -0.28, -0.15 | 8.11E-11 | -0.13 | -0.20, -0.07 | 1.13E-04 | -0.18 | -0.24, -0.11 | 9.31E-08 |
|  | CV of ITI | -0.14 | -0.20, -0.07 | 4.18E-05 | -0.11 | -0.17, -0.04 | 1.73E-03 | -0.12 | -0.18, -0.05 | 4.71E-04 |
|  | Skewness of ITI | 0.00 | -0.07, 0.06 | 9.47E-01 | 0.00 | -0.06, 0.07 | 9.82E-01 | -0.02 | -0.09, 0.04 | 4.68E-01 |
|  | Kurtosis of ITI | 0.03 | -0.04, 0.09 | 3.83E-01 | 0.02 | -0.04, 0.09 | 5.33E-01 | 0.03 | -0.04, 0.09 | 4.01E-01 |
|  | Alternating Tap Ratio | 0.05 | -0.01, 0.12 | 1.07E-01 | 0.07 | -0.00, 0.13 | 5.39E-02 | 0.04 | -0.03, 0.10 | 2.52E-01 |
|  | Mean of Left ITI | -0.22 | -0.29, -0.16 | 3.21E-11 | -0.15 | -0.22, -0.08 | 1.15E-05 | -0.18 | -0.25, -0.12 | 1.03E-07 |
|  | SD of Left ITI | -0.18 | -0.25, -0.11 | 1.48E-07 | -0.13 | -0.20, -0.07 | 1.07E-04 | -0.16 | -0.22, -0.10 | 1.11E-06 |
|  | Mean of Right ITI | -0.20 | -0.27, -0.14 | 2.40E-09 | -0.18 | -0.24, -0.11 | 2.28E-07 | -0.18 | -0.25, -0.11 | 1.16E-07 |
|  | SD of Right ITI | -0.12 | -0.19, -0.05 | 6.16E-04 | -0.10 | -0.16, -0.03 | 6.63E-03 | -0.11 | -0.18, -0.05 | 6.70E-04 |
|  | ITI Range | -0.23 | -0.30, -0.17 | 6.21E-12 | -0.16 | -0.22, -0.09 | 5.60E-06 | -0.18 | -0.25, -0.12 | 2.82E-08 |
|  | ITI Slope | -0.11 | -0.17, -0.05 | 7.48E-04 | 0.06 | -0.01, 0.12 | 9.18E-02 | -0.04 | -0.10, 0.02 | 2.13E-01 |
|  | Last to First ITI | -0.06 | -0.13, 0.00 | 5.40E-02 | -0.05 | -0.11, 0.02 | 1.34E-01 | -0.02 | -0.09, 0.04 | 5.09E-01 |
|  | Micro Fluctuation Index | -0.16 | -0.22, -0.09 | 2.91E-06 | -0.13 | -0.20, -0.07 | 9.03E-05 | -0.15 | -0.21, -0.08 | 8.31E-06 |

Significant associations were claimed if *P*<0.05/15=0.0033.

**Supplemental Table 6**. The most significant digital finger tapping feature for each cognitive domain based on trials with Alternating Tap Ratio ≥0.8.

| **Cognitive domain** | **Number of significant finger tapping features** | **Most significant association** | | | |
| --- | --- | --- | --- | --- | --- |
|  |  | **Finger tapping feature** | **β** | **95% CI** | **P value** |
| Memory | 9 | Mean ITI | -0.18 | -0.26, -0.11 | <.001 |
| Executive function | 12 | Mean ITI | -0.27 | -0.33, -0.20 | <.001 |
| Language | 10 | Mean of Left ITI | -0.20 | -0.28, -0.12 | <.001 |
| Visuospatial function | 7 | Mean ITI | -0.26 | -0.37, -0.14 | <.001 |
| Global cognitive function | 12 | Mean ITI | -0.23 | -0.29, -0.16 | <.001 |

Significant associations were claimed if *P*<0.05/15=0.0033.

**Supplemental Table 7**. The most significant digital finger tapping feature for each cognitive domain based on the first three sessions.

| **Cognitive domain** | **Number of significant finger tapping features** | **Most significant association** | | | |
| --- | --- | --- | --- | --- | --- |
|  |  | **Finger tapping feature** | **β** | **95% CI** | ***P* value** |
| Memory | 3 | Mean ITI | -0.12 | -0.19, -0.05 | .001 |
| Executive function | 10 | Mean ITI | -0.19 | -0.26, -0.12 | <.001 |
| Language | 5 | Mean of Right ITI | -0.17 | -0.26, -0.09 | <.001 |
| Visuospatial function | 1 | CV of ITI | -0.19 | -0.31, -0.06 | .003 |
| Global cognitive function | 8 | Mean ITI | -0.16 | -0.23, -0.09 | <.001 |

Significant associations were claimed if *P*<0.05/15=0.0033.

**Supplemental Table 8.** The most significant interaction terms for each cognitive domain.

| **Cognitive domain** | **Number of significant interactions** | **Most significant interaction** | | | |
| --- | --- | --- | --- | --- | --- |
|  |  | **Finger tapping feature x cohort*** | **β** | **95% CI** | **P value** |
| Memory | 0 | - | - | - | - |
| Executive function | 4 | ITI Range | 0.23 | 0.07, 0.38 | 0.01 |
| Language | 3 | ITI Slope | 0.48 | 0.07, 0.90 | 0.02 |
| Visuospatial function | 0 | - | - | - | - |
| Global cognitive function | 3 | ITI Slope | 0.38 | 0.04, 0.71 | 0.03 |

*Offspring cohort was used as reference group.

Interaction terms were significant at *P* < 0.05. For executive function, significant interactions involve SD of ITI, CV of ITI, SD of right ITI, and ITI range. For language, they involve number of taps, SD of left ITI, and ITI slope. For global cognitive function, they involve number of taps, SD of left ITI, and ITI slope.

**Supplemental Table 9**. The association between digital finger tapping features and cognitive domains based on participants with complete NP test data.

| **Cognitive domain** | **Finger tapping feature** | **β** | **95% CI** | ***P* value** |
| --- | --- | --- | --- | --- |
| Memory | Number of taps | 0.06 | -0.02, 0.15 | 1.55E-01 |
|  | Mean ITI | -0.08 | -0.17, 0.01 | 7.24E-02 |
|  | SD of ITI | -0.11 | -0.19, -0.02 | 1.23E-02 |
|  | CV of ITI | -0.10 | -0.19, -0.02 | 1.55E-02 |
|  | Skewness of ITI | -0.07 | -0.15, 0.02 | 1.16E-01 |
|  | Kurtosis of ITI | -0.06 | -0.14, 0.02 | 1.53E-01 |
|  | Alternating Tap Ratio | 0.04 | -0.05, 0.12 | 3.60E-01 |
|  | Mean of Left ITI | -0.08 | -0.17, 0.01 | 7.06E-02 |
|  | SD of Left ITI | -0.10 | -0.18, -0.01 | 2.61E-02 |
|  | Mean of Right ITI | -0.09 | -0.18, -0.01 | 3.48E-02 |
|  | SD of Right ITI | -0.11 | -0.20, -0.03 | 1.01E-02 |
|  | ITI Range | -0.13 | -0.22, -0.05 | 2.83E-03 |
|  | ITI Slope | 0.11 | 0.02, 0.19 | 1.56E-02 |
|  | Last to First ITI | 0.00 | -0.08, 0.08 | 9.92E-01 |
|  | Micro Fluctuation Index | -0.11 | -0.19, -0.02 | 1.23E-02 |
| Executive function | Number of taps | 0.15 | 0.06, 0.24 | 1.53E-03 |
|  | Mean ITI | -0.20 | -0.29, -0.11 | 1.48E-05 |
|  | SD of ITI | -0.16 | -0.25, -0.08 | 2.48E-04 |
|  | CV of ITI | -0.07 | -0.16, 0.02 | 1.13E-01 |
|  | Skewness of ITI | -0.05 | -0.13, 0.04 | 2.82E-01 |
|  | Kurtosis of ITI | 0.01 | -0.08, 0.09 | 9.05E-01 |
|  | Alternating Tap Ratio | 0.05 | -0.04, 0.14 | 2.88E-01 |
|  | Mean of Left ITI | -0.19 | -0.28, -0.10 | 2.60E-05 |
|  | SD of Left ITI | -0.21 | -0.30, -0.13 | 1.55E-06 |
|  | Mean of Right ITI | -0.20 | -0.29, -0.11 | 1.12E-05 |
|  | SD of Right ITI | -0.12 | -0.21, -0.03 | 8.02E-03 |
|  | ITI Range | -0.19 | -0.28, -0.11 | 1.41E-05 |
|  | ITI Slope | 0.01 | -0.08, 0.10 | 7.50E-01 |
|  | Last to First ITI | 0.00 | -0.08, 0.09 | 9.61E-01 |
|  | Micro Fluctuation Index | -0.17 | -0.26, -0.09 | 8.44E-05 |
| Language | Number of taps | 0.14 | 0.04, 0.25 | 6.51E-03 |
|  | Mean ITI | -0.20 | -0.30, -0.10 | 1.22E-04 |
|  | SD of ITI | -0.18 | -0.28, -0.08 | 3.35E-04 |
|  | CV of ITI | -0.08 | -0.18, 0.02 | 1.22E-01 |
|  | Skewness of ITI | -0.06 | -0.16, 0.04 | 2.24E-01 |
|  | Kurtosis of ITI | -0.01 | -0.11, 0.09 | 8.65E-01 |
|  | Alternating Tap Ratio | -0.05 | -0.15, 0.05 | 3.49E-01 |
|  | Mean of Left ITI | -0.22 | -0.32, -0.12 | 2.93E-05 |
|  | SD of Left ITI | -0.20 | -0.30, -0.10 | 1.10E-04 |
|  | Mean of Right ITI | -0.20 | -0.30, -0.10 | 1.30E-04 |
|  | SD of Right ITI | -0.11 | -0.21, -0.01 | 3.48E-02 |
|  | ITI Range | -0.18 | -0.28, -0.08 | 4.10E-04 |
|  | ITI Slope | 0.04 | -0.07, 0.14 | 4.83E-01 |
|  | Last to First ITI | -0.04 | -0.13, 0.06 | 4.65E-01 |
|  | Micro Fluctuation Index | -0.19 | -0.29, -0.09 | 2.07E-04 |
| Visuospatial | Number of taps | 0.06 | -0.09, 0.21 | 4.23E-01 |
|  | Mean ITI | -0.13 | -0.28, 0.02 | 8.54E-02 |
|  | SD of ITI | -0.13 | -0.27, 0.02 | 8.78E-02 |
|  | CV of ITI | -0.08 | -0.22, 0.07 | 2.88E-01 |
|  | Skewness of ITI | -0.07 | -0.22, 0.07 | 2.99E-01 |
|  | Kurtosis of ITI | -0.05 | -0.19, 0.09 | 4.82E-01 |
|  | Alternating Tap Ratio | 0.03 | -0.11, 0.18 | 6.43E-01 |
|  | Mean of Left ITI | -0.15 | -0.30, -0.00 | 4.89E-02 |
|  | SD of Left ITI | -0.16 | -0.31, -0.02 | 2.98E-02 |
|  | Mean of Right ITI | -0.11 | -0.26, 0.04 | 1.52E-01 |
|  | SD of Right ITI | -0.06 | -0.21, 0.09 | 4.44E-01 |
|  | ITI Range | -0.13 | -0.28, 0.02 | 8.25E-02 |
|  | ITI Slope | -0.06 | -0.21, 0.08 | 4.02E-01 |
|  | Last to First ITI | -0.10 | -0.24, 0.04 | 1.75E-01 |
|  | Micro Fluctuation Index | -0.12 | -0.27, 0.02 | 1.02E-01 |
| Global cognitive function | Number of taps | 0.10 | 0.03, 0.18 | 6.83E-03 |
|  | Mean ITI | -0.15 | -0.23, -0.08 | 5.74E-05 |
|  | SD of ITI | -0.15 | -0.22, -0.07 | 8.04E-05 |
|  | CV of ITI | -0.08 | -0.16, -0.01 | 2.48E-02 |
|  | Skewness of ITI | -0.06 | -0.13, 0.01 | 8.64E-02 |
|  | Kurtosis of ITI | -0.03 | -0.10, 0.04 | 4.34E-01 |
|  | Alternating Tap Ratio | 0.02 | -0.05, 0.09 | 6.23E-01 |
|  | Mean of Left ITI | -0.16 | -0.23, -0.09 | 2.27E-05 |
|  | SD of Left ITI | -0.17 | -0.24, -0.10 | 5.52E-06 |
|  | Mean of Right ITI | -0.15 | -0.22, -0.08 | 6.79E-05 |
|  | SD of Right ITI | -0.10 | -0.18, -0.03 | 7.84E-03 |
|  | ITI Range | -0.16 | -0.23, -0.09 | 1.78E-05 |
|  | ITI Slope | 0.02 | -0.05, 0.10 | 5.33E-01 |
|  | Last to First ITI | -0.03 | -0.10, 0.04 | 3.68E-01 |
|  | Micro Fluctuation Index | -0.15 | -0.22, -0.08 | 5.59E-05 |

Significant associations were claimed if *P*<0.05/15=0.0033.

**Supplemental Table 10**. The most significant digital finger tapping feature for each cognitive domain based on left-hand administered tests

| **Cognitive domain** | **Number of significant finger tapping features** | **Most significant association** | | | |
| --- | --- | --- | --- | --- | --- |
|  |  | **Finger tapping features** | **β** | **95% CI** | **P value** |
| Memory | 5 | Mean of Right ITI | -0.15 | -0.22, -0.07 | <.001 |
| Executive function | 10 | Mean of Right ITI | -0.19 | -0.26, -0.12 | <.001 |
| Language | 8 | Mean of Right ITI | -0.19 | -0.27, -0.11 | <.001 |
| Visuospatial function | 1 | Mean of Right ITI | -0.17 | -0.29, -0.06 | 0.003 |
| Global cognitive function | 9 | Mean of Right ITI | -0.18 | -0.24, -0.11 | <.001 |

Significant associations were claimed if *P*<0.05/15=0.0033.

**Supplemental Table 11**. The most significant digital finger tapping feature for each cognitive domain based on right-hand administered tests

| **Cognitive domain** | **Number of significant finger tapping features** | **Most significant association** | | | |
| --- | --- | --- | --- | --- | --- |
|  |  | **Finger tapping features** | **β** | **95% CI** | **P value** |
| Memory | 9 | ITI Range | -0.16 | -0.23, -0.09 | <.001 |
| Executive function | 8 | Mean ITI | -0.18 | -0.25, -0.12 | <.001 |
| Language | 8 | Mean of Right ITI | -0.22 | -0.30, -0.13 | <.001 |
| Visuospatial function | 6 | SD of ITI | -0.23 | -0.34, -0.11 | <.001 |
| Global cognitive function | 10 | ITI Range | -0.18 | -0.25, -0.12 | <.001 |

Significant associations were claimed if *P*<0.05/15=0.0033.

**Supplemental Table 12**. The most significant digital finger tapping feature for each cognitive domain among right-handed participants.

| **Cognitive domain** | **Number of significant finger tapping features** | **Most significant association** | | | |
| --- | --- | --- | --- | --- | --- |
|  |  | **Finger tapping features** | **β** | **95% CI** | **P value** |
| Memory | 9 | ITI Range | -0.20 | -0.27, -0.12 | <.001 |
| Executive function | 11 | Mean ITI | -0.28 | -0.35, -0.21 | <.001 |
| Language | 9 | Mean of Right ITI | -0.26 | -0.35, -0.18 | <.001 |
| Visuospatial function | 8 | ITI Range | -0.29 | -0.42, -0.16 | <.001 |
| Global cognitive function | 11 | Mean ITI | -0.25 | -0.31, -0.18 | <.001 |

Significant associations were claimed if *P*<0.05/15=0.0033.

**Supplemental Table 13.** The most significant digital finger tapping feature for each cognitive domain based on left hand administered tests among right-handed participants.

| **Cognitive domain** | **Number of significant finger tapping features** | **Most significant association** | | | |
| --- | --- | --- | --- | --- | --- |
|  |  | **Finger tapping features** | **β** | **95% CI** | **P value** |
| Memory | 5 | Mean of Right ITI | -0.14 | -0.22, -0.07 | <.001 |
| Executive function | 10 | Mean of Right ITI | -0.19 | -0.27, -0.12 | <.001 |
| Language | 8 | Mean of Right ITI | -0.20 | -0.28, -0.12 | <.001 |
| Visuospatial function | 2 | ITI Range | -0.22 | -0.34, -0.09 | <.001 |
| Global cognitive function | 9 | Mean of Right ITI | -0.18 | -0.25, -0.11 | <.001 |

Significant associations were claimed if *P*<0.05/15=0.0033.

**Supplemental Table 14**. The most significant digital finger tapping feature for each cognitive domain based on right hand administered tests among right-handed participants.

| **Cognitive domain** | **Number of significant finger tapping features** | **Most significant association** | | | |
| --- | --- | --- | --- | --- | --- |
|  |  | **Finger tapping features** | **β** | **95% CI** | **P value** |
| Memory | 7 | ITI Range | -0.15 | -0.22, -0.07 | <.001 |
| Executive function | 7 | Mean ITI | -0.18 | -0.25, -0.10 | <.001 |
| Language | 8 | Mean of Right ITI | -0.23 | -0.32, -0.15 | <.001 |
| Visuospatial function | 6 | Mean ITI | -0.24 | -0.37, -0.12 | <.001 |
| Global cognitive function | 9 | Mean ITI | -0.19 | -0.26, -0.12 | <.001 |

Significant associations were claimed if *P*<0.05/15=0.0033.

**Supplemental Table 15.** The most significant digital finger tapping feature for each cognitive domain based on dominant hand administered tests.

| **Cognitive domain** | **Number of significant finger tapping features** | **Most significant association** | | | |
| --- | --- | --- | --- | --- | --- |
|  |  | **Finger tapping features** | **β** | **95% CI** | **P value** |
| Memory | 6 | Mean of Left ITI | -0.14 | -0.22, -0.06 | <.001 |
| Executive function | 8 | Mean ITI | -0.19 | -0.26, -0.12 | <.001 |
| Language | 7 | Mean of Right ITI | -0.22 | -0.31, -0.14 | <.001 |
| Visuospatial function | 4 | Mean ITI | -0.26 | -0.38, -0.14 | <.001 |
| Global cognitive function | 9 | Mean ITI | -0.20 | -0.27, -0.13 | <.001 |

Significant associations were claimed if *P*<0.05/15=0.0033.

**Supplemental Table 16.** The most significant digital finger tapping feature for each cognitive domain based on non-dominant hand administered tests.

| **Cognitive domain** | **Number of significant finger tapping features** | **Most significant association** | | | |
| --- | --- | --- | --- | --- | --- |
|  |  | **Finger tapping features** | **β** | **95% CI** | **P value** |
| Memory | 5 | Mean of Right ITI | -0.15 | -0.22, -0.07 | <.001 |
| Executive function | 10 | Mean of Right ITI | -0.20 | -0.27, -0.13 | <.001 |
| Language | 8 | Mean of Right ITI | -0.19 | -0.27, -0.11 | <.001 |
| Visuospatial function | 5 | ITI Range | -0.23 | -0.35, -0.11 | <.001 |
| Global cognitive function | 9 | ITI Range | -0.18 | -0.25, -0.12 | <.001 |

Significant associations were claimed if *P*<0.05/15=0.0033.

**Supplemental Figure 1.** Screenshot of the Two Finger Tap application

**
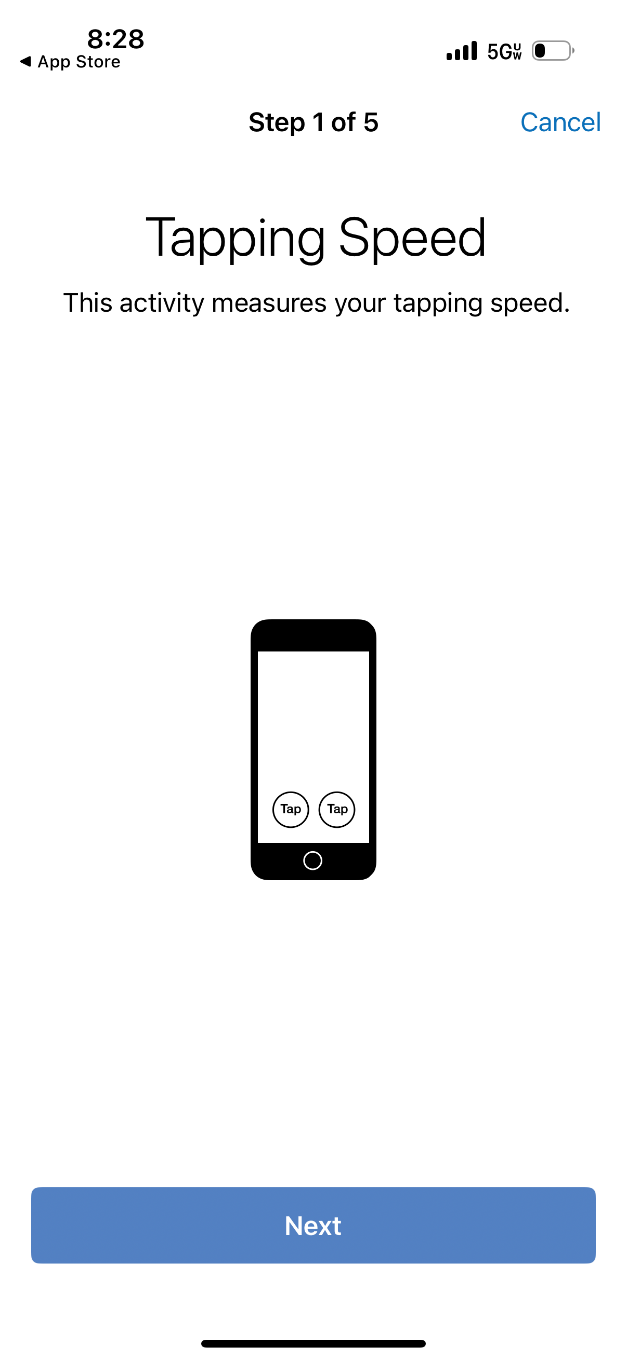

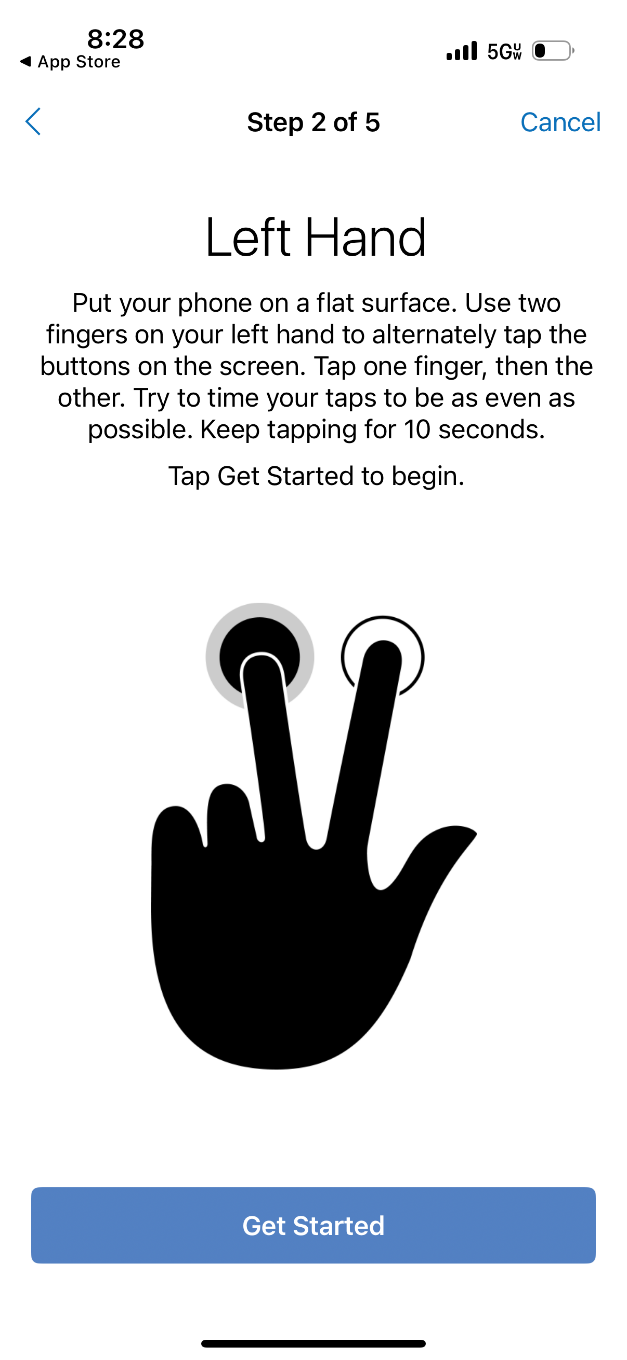

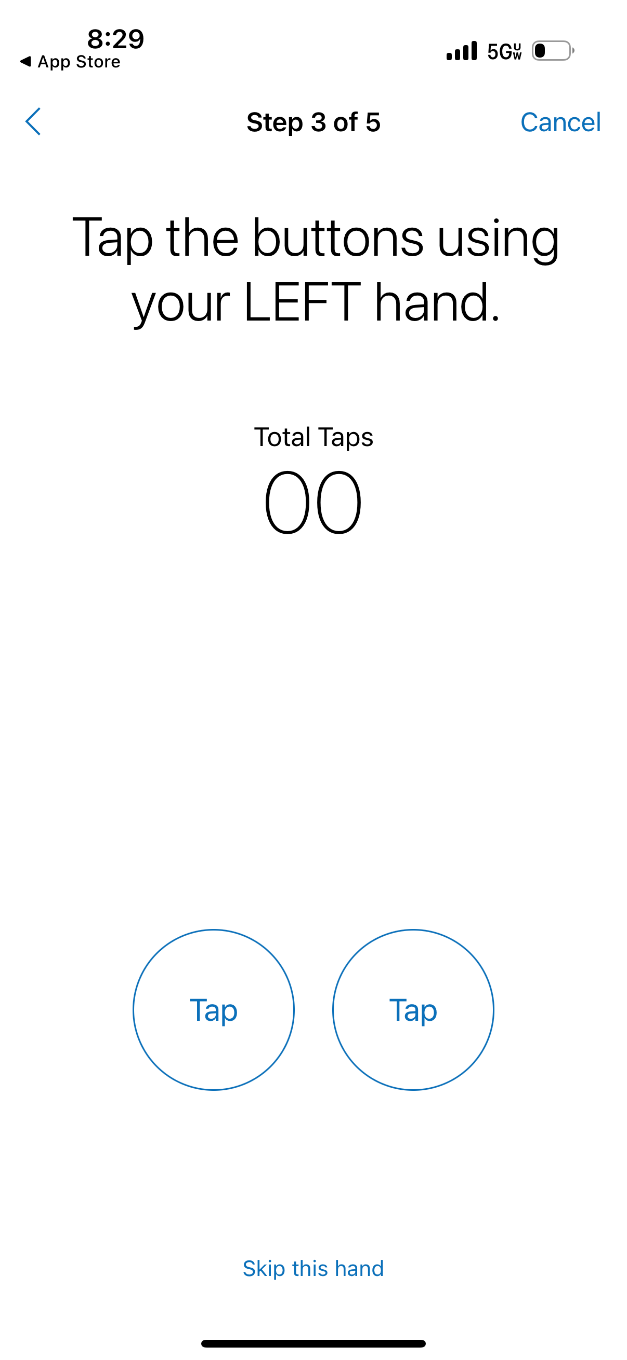
**

**
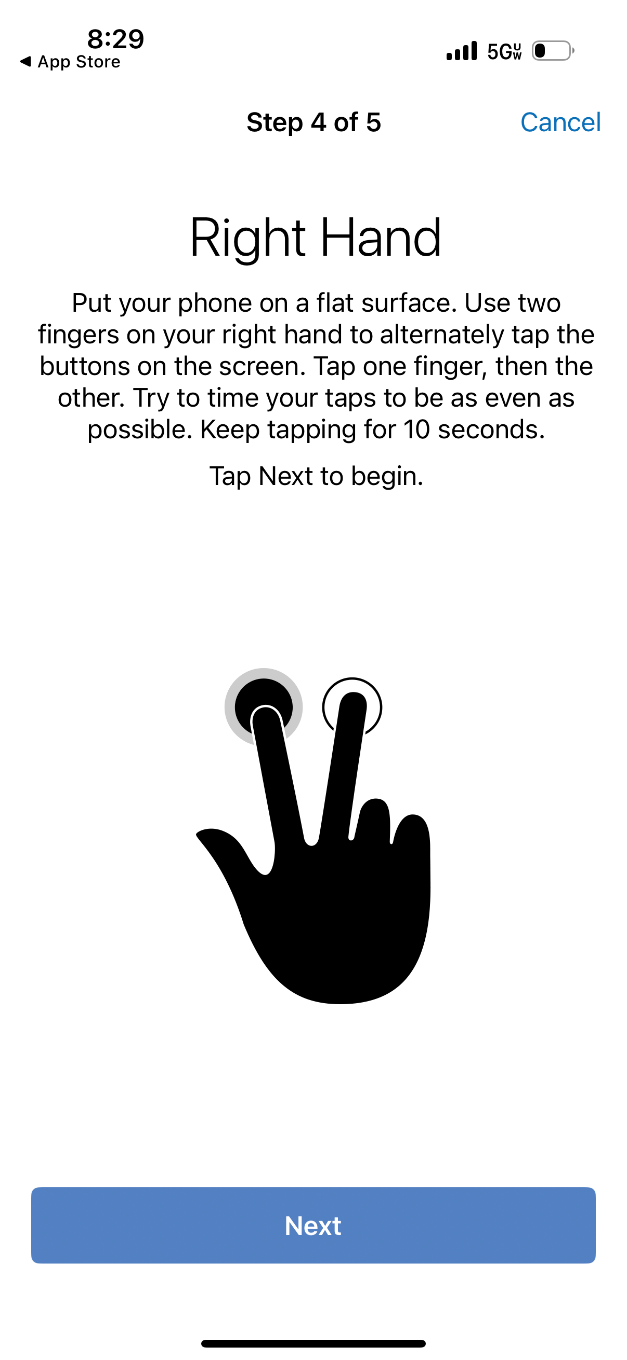

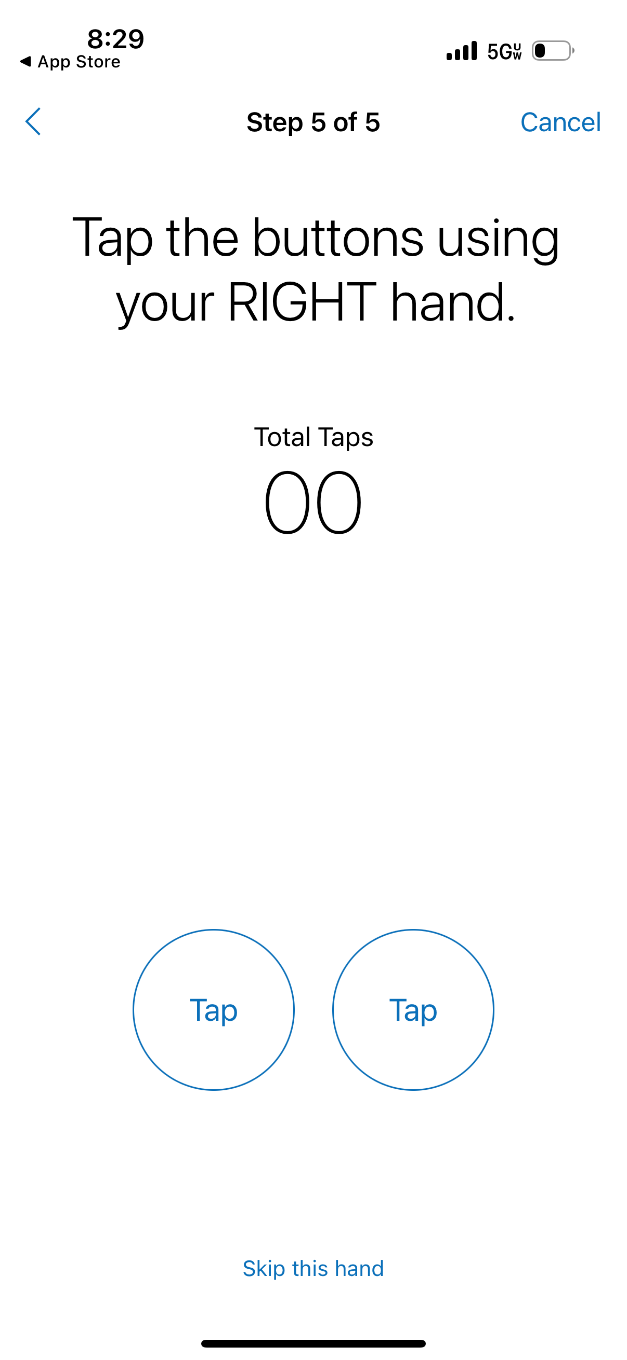

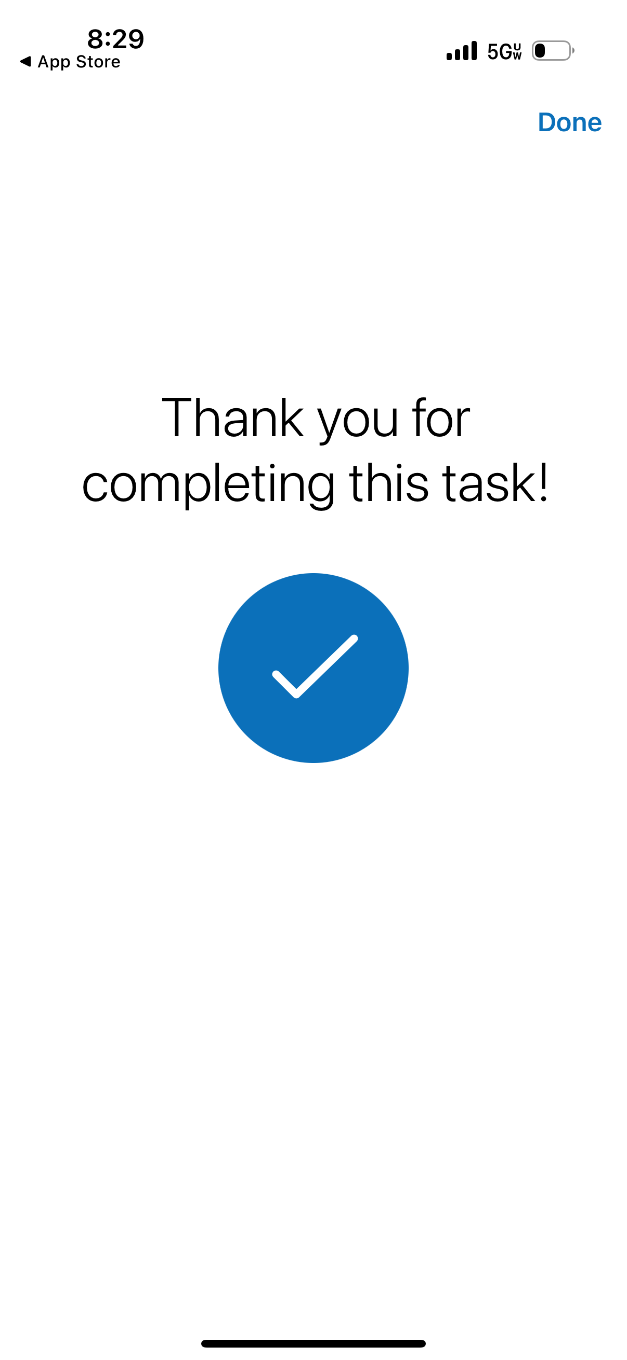
**

**Supplemental Figure 2.** Examples of finger tapping test
